# CAG and GGC repeat polymorphisms in the androgen receptor gene and breast cancer susceptibility in *BRCA1/2* carriers and non-carriers

**DOI:** 10.1054/bjoc.2001.1777

**Published:** 2001-07

**Authors:** L Kadouri, D F Easton, S Edwards, A Hubert, Z Kote-Jarai, B Glaser, F Durocher, D Abeliovich, T Peretz, R A Eeles

**Affiliations:** Sharett Institute of Oncology, Hebrew University-Hadassah Medical Center, Jerusalem, Israel; CRC Section of Cancer Genetics, The Institute of Cancer Research, Sutton, UK; CRC Genetic Epidemiology Unit, Strangeways Research Laboratories, Cambridge, UK; Department of Endocrinology and Metabolism, Hebrew University-Hadassah Medical Center, Jerusalem, Israel; Human Genetic Laboratories, Hebrew University-Hadassah Medical Center, Jerusalem, Israel; The Royal Marsden NHS Trust, Sutton, UK

**Keywords:** *BRCA1/2*, androgen receptor, polymorphisms, breast cancer risk

## Abstract

Variation in the penetrance estimates for *BRCA1* and *BRCA2* mutations carriers suggests that other genetic polymorphisms may modify the cancer risk in carriers. A previous study has suggested that *BRCA1* carriers with longer lengths of the CAG repeat in the androgen receptor (*AR*) gene are at increased risk of breast cancer (BC). We genotyped 188 *BRCA1/2* carriers (122 affected and 66 unaffected with breast cancer), 158 of them of Ashkenazi origin, 166 BC cases without *BRCA1/2* mutations and 156 Ashkenazi control individuals aged over 56 for the *AR* CAG and GGC repeats. In carriers, risk analyses were conducted using a variant of the log-rank test, assuming two sets of risk estimates in carriers: penetrance estimates based on the Breast Cancer Linkage Consortium (BCLC) studies of multiple case families, and lower estimates as suggested by population-based studies. We found no association of the CAG and GGC repeats with BC risk in either *BRCA1/2* carriers or in the general population. Assuming *BRCA1/2* penetrance estimates appropriate to the Ashkenazi population, the estimated RR per repeat adjusted for ethnic group (Ashkenazi and non-Ashkenazi) was 1.05 (95%CI 0.97–1.17) for BC and 1.00 (95%CI 0.83–1.20) for ovarian cancer (OC) for CAG repeats and 0.96 (95%CI 0.80–1.15) and 0.90 (95%CI 0.60–1.22) respectively for GGC repeats. The corresponding RR estimates for the unselected case–control series were 1.00 (95%CI 0.91–1.10) for the CAG and 1.05 (95%CI 0.90–1.22) for the GGC repeats. The estimated relative risk of BC in carriers associated with ≥28 CAG repeats was 1.08 (95%CI 0.45–2.61). Furthermore, no significant association was found if attention was restricted to the Ashkenazi carriers, or only to *BRCA1* or *BRCA2* carriers. We conclude that, in contrast to previous observations, if there is any effect of the AR repeat length on *BRCA1* penetrance, it is likely to be weak. © 2001 Cancer Research Campaign http://www.bjcancer.com


*British Journal of Cancer* (2001) **85**(1), 36–40
© 2001 Cancer Research Campaign
doi: 10.1054/ bjoc.2001.1777, available online at http://www.idealibrary.com on IDEAL®

### CAG and GGC repeat polymorphisms in the androgen receptor gene and breast cancer susceptibility in *BRCA1/2* carriers and non-carriers

**L Kadouri[1,2], DF Easton[3], S Edwards[2], A Hubert[1], Z Kote-Jarai[2], B Glaser[4], F Durocher[3], D Abeliovich[5], T Peretz[1] and RA Eeles[2,6]**

[1]Sharett Institute of Oncology, Hebrew University-Hadassah Medical Center, Jerusalem, Israel; [2]CRC Section of Cancer Genetics, The Institute of Cancer Research, Sutton, UK; [3]CRC Genetic Epidemiology Unit, Strangeways Research Laboratories, Cambridge, UK; [4]Department of Endocrinology and Metabolism, Hebrew University-Hadassah Medical Center, Jerusalem Israel; [5]Human Genetic Laboratories, Hebrew University-Hadassah Medical Center, Jerusalem, Israel; [6]The Royal Marsden NHS Trust, Sutton, UK

**Summary** Variation in the penetrance estimates for *BRCA1* and *BRCA2* mutations carriers suggests that other genetic polymorphisms may modify the cancer risk in carriers. A previous study has suggested that *BRCA1* carriers with longer lengths of the CAG repeat in the androgen receptor (*AR*) gene are at increased risk of breast cancer (BC). We genotyped 188 *BRCA1/2* carriers (122 affected and 66 unaffected with breast cancer), 158 of them of Ashkenazi origin, 166 BC cases without *BRCA1/2* mutations and 156 Ashkenazi control individuals aged over 56 for the *AR* CAG and GGC repeats. In carriers, risk analyses were conducted using a variant of the log-rank test, assuming two sets of risk estimates in carriers: penetrance estimates based on the Breast Cancer Linkage Consortium (BCLC) studies of multiple case families, and lower estimates as suggested by population-based studies. We found no association of the CAG and GGC repeats with BC risk in either *BRCA1/2* carriers or in the general population. Assuming *BRCA1/2* penetrance estimates appropriate to the Ashkenazi population, the estimated RR per repeat adjusted for ethnic group (Ashkenazi and non-Ashkenazi) was 1.05 (95%CI 0.97–1.17) for BC and 1.00 (95%CI 0.83–1.20) for ovarian cancer (OC) for CAG repeats and 0.96 (95%CI 0.80–1.15) and 0.90 (95%CI 0.60–1.22) respectively for GGC repeats. The corresponding RR estimates for the unselected case–control series were 1.00 (95%CI 0.91–1.10) for the CAG and 1.05 (95%CI 0.90–1.22) for the GGC repeats. The estimated relative risk of BC in carriers associated with ≥28 CAG repeats was 1.08 (95%CI 0.45–2.61). Furthermore, no significant association was found if attention was restricted to the Ashkenazi carriers, or only to *BRCA1* or *BRCA2* carriers. We conclude that, in contrast to previous observations, if there is any effect of the AR repeat length on *BRCA1* penetrance, it is likely to be weak. © 2001 Cancer Research Campaign http://www.bjcancer.com

**Keywords**: *BRCA1/2*; androgen receptor; polymorphisms; breast cancer risk

Carriers of germline mutations in *BRCA1* or *BRCA2* are at substantially increased risk of both breast (BC) and ovarian cancer (OC). There are, however, significant differences in the penetrance estimates generated by different studies. Studies by the Breast Cancer Linkage Consortium (BCLC) have estimated that cumulative risks of BC and OC, by age 70, are approximately 70% and 40%, respectively, in *BRCA1* carriers, and 70% and 27% in *BRCA2* carriers (Easton et al, 1995; Ford et al, 1998). In contrast, risk estimates based on relatives of unselected BC and OC patients with mutations have generally been somewhat lower (Streuwing et al, 1997; Thorlacius et al, 1998; Peto et al, 1999). Although some of this difference may be due to differences in risk conferred by different mutations, it is likely that modification of risk by other genes or environmental risk factors clustering in families explain most of this difference. It is also possible that these modifying genes are associated with variation in risk in non-carriers.

Two population-based case–control studies, based on 368 BC cases diagnosed below age 40 (Spurdle et al, 1999) and 508 BC cases diagnosed below age 55 (Dunning et al, 1999) respectively, have examined the association between CAG repeat length and BC risk, and neither found any evidence of an interaction. However, a recent study of 304 *BRCA1* carriers by Rebbeck et al (1999) found that longer repeat lengths, particularly genotypes with ≥28 repeats, were associated with a higher risk of breast cancer.

In an attempt to replicate this latter observation, we have genotyped the AR repeats in a large series of *BRCA1/2* carriers, mainly from an Ashkenazi Jewish study in Israel. We also studied an unselected series of Ashkenazi BC cases and controls for comparison. This population has a strong founder effect such that 3 mutations, the 185delAG and 5382insC mutations in *BRCA1* and the 617delT mutation in *BRCA2*, account for almost all the *BRCA1/2* carriers in this population and have a combined population frequency of about 2.5% (Struewing et al, 1997).

### SUBJECTS AND METHODS

### Study population

Two populations were studied; *BRCA1/2* carriers and non-carrier BC patients and controls. Blood samples from 188 *BRCA1/2* carriers were identified through two centres: 142 were collected

*Received 22 August 2000*
*Revised 12 February 2001*
*Accepted 12 February 2001*

*Correspondence to:* RA Eeles through the oncology department and the cancer genetic clinic in Hadassah Medical Centre in Jerusalem, Israel and 46 through the cancer genetic clinic in the Royal Marsden NHS Trust, London, UK. Cases were tested on the basis of a family history of breast and/or ovarian cancer or on the basis of their Ashkenazi origin. The cases from Jerusalem were all but one carriers of one of the Ashkenazi founder mutations (79: 185delAG, 20: 5382insC in *BRCA1* and 42: 6174delT in *BRCA2*). The UK carriers included 17 Ashkenazis (one individual carried both a 185delAG and 6174delT) and 29 other mutations (22 *BRCA1*; 7 *BRCA2*). Of the 188 carriers, 110 were affected with breast cancer, 26 with ovarian cancer and 14 with both cancers. 36 were unaffected. The characteristics of the carrier series are summarized in Table 1.

A further 166 Ashkenazi Jewish BC patients were studied. They were ascertained through the oncology department in Hadassah over the period 1994–98 in the same way as the *BRCA1/2* carriers but were not found to carry any of the 3 Ashkenazi founder *BRCA1/2* mutations. 24 (14%) of the patients were diagnosed below age 40, 54 (33%) aged 40–49 and 88 (53%) aged 50+ years. Of them, 71/111 (64%) reported a positive family history of breast cancer, data were not available on the rest. Thus, this series has a slightly higher frequency of early onset cases and cases with a family history than would be expected in the whole population.

The controls were 152 females who took part in an independent study into genetics of diabetes. All controls were cancer-free, from Ashkenazi origin and aged above 56 years (mean 69, range 56–92 years). All participants signed an informed consent approved by the institutional ethics committee.

### Genotyping methods

Genomic DNA was extracted according to standard protocols, and used as a template for PCR as previously described (Edwards et al, 1999). Primer sequences were 5′-TCCGCGAAGTGATCCA-GAAC and 5′-CTTGGGGAGAACCATCCTCA for the CAG repeat, and 5′-TCCTGGCACACTCTCTTCAC and 5′-GCCAGGGTACCACACATCAGGT for the GGC repeat. The forward primers were labelled by T4-PNK enzyme and $^{32}$P-dATP. PCR reactions were conducted using Amplitaq Gold (Perkin-Elmar) according to the manufacturer's recommended conditions using 0.8 and 1.3 mM $MgCl_2$ for the CAG and GGC amplification respectively. For the GGC reaction a 3:1 mixture of 7-deaza-dGTP:dGTP and 2.5% v/v DMSO (Sigma, Poole, UK: molecular grade) were used. Thermocycling conditions for the CAG amplification were 95˚C for 9 min, 1 min at 94˚C, 30 sec at 64˚C and 1 min at 72˚C for 4 cycles, followed by a 'touchdown' reduction of annealing temperature by 2˚C every 4 cycles until 54˚C, than another 29 cycles at 54˚C annealing temperature. For the GGC repeat a computed touchdown algorithm was used starting with 64˚C and ending at 54˚C, which is equivalent to a reduction of 0.5˚C per cycle. A further 25 cycles were conducted using an annealing temperature of 54˚C.

PCR products were analysed by electrophoresis in denaturing 6% polyacrylamide gels. For scoring, a ladder of samples with 15, 17, 19, 21, 23, 25, 27 CAG repeats and a sample with 16 GGC repeats were run at regular intervals.

### Statistical analysis

6 of the 188 carriers were excluded from all analyses: 2 for whom the date of birth was unknown, and 4 individuals recorded as affected but for whom the age at diagnosis was unknown. A further 4 individuals could not be scored for the CAG repeat and one individual for the GGC repeat, so the final analyses of the CAG and GGC repeats were based on 178 and 181 individuals, respectively.

Separate analyses were performed for breast and ovarian cancer risk. For simplicity the analyses were based on the occurrence of the first cancer (i.e. all individuals were censored at the first cancer). With this classification there were 118 breast cancer cases and 29 ovarian cancer cases (including one woman diagnosed simultaneously with both cancers and therefore counted as affected with both cancers) and 36 unaffected carriers.

The effects of AR genotypes on breast cancer risk in mutation carriers were assessed using a variant of the log-rank test, based on the statistic:

$$U = \sum_{jk} \Sigma\, (z_{jk} - \bar{z}_j)(O_{jk} - E_{jk})$$

Since the distributions of age and disease status, and also genotype distributions, were different in the Ashkenazi and non-Ashkenazi populations, these were treated as separate strata in the analysis. In the above formula, $O_{jk}$ is the disease status of individual k in stratum j, $E_{jk}$ is the expected cumulative incidence rate of breast (or ovarian) cancer for the same individual, given their age and the fact that they were a mutation carrier, and $z_{jk}$ is their value of the covariate. $\bar{z}_j$ is the mean value of the covariate in stratum j. The significance level for U was obtained by simulation, by permuting genotypes randomly among individuals within the same stratum. A total of 10 000 replicates were used. This test is the locally most powerful test for detecting an increased relative risk (constant over age), assuming that the overall incidence rates in carriers are accurately specified (D Easton, in preparation). To guard against loss of power due to misspecification of the incidence rates, the analyses were performed under two assumptions: (a) with the expected incidence rates based on the BCLC studies of 'high-risk' *BRCA1* and *BRCA2* families with numerous affecteds (Easton et al, 1995; Ford et al, 1998), and (b) with expected incidence rates half of the BCLC incidence rates. Under assumption (b), the cumulative risk of breast cancer by age 70 would be 0.47 in *BRCA1* carriers and 0.62 in *BRCA2* carriers, similar to estimates found in some of the population studies (Streuwing et al, 1997; Thorlacius et al, 1998; Peto et al, 1999). Since most of the carriers in this study were ascertained on the basis of some degree of family history, it is likely that the appropriate incidence rates fall somewhere between these two values.

An estimate of the relative risk is provided by exp (–U/V), with the variance of the log-relative risk given by 1/V, where V is the variance of U.

For AR, the average number of repeats was first analysed as a continuous covariate. We also performed a specific test of the hypothesis suggested by Rebbeck et al (1999), that carriers with ≥28 repeats are at increased risk of breast cancer. As a further check we also analysed our data using Cox regression, as if the individuals in the study represented a cohort of carriers. This analysis is not strictly justified since the individuals are selected to an extent based on their disease status, and it cannot therefore provide an unbiased estimate of the relative risk (though it should still provide a valid significance test). We performed this analysis for direct comparability with the report of Rebbeck et al (1999) who also used this approach on a similar data set.

*© 2001 Cancer Research Campaign*

The case–control analyses based on the non-carrier BC cases ($n$ = 166) and controls ($n$ = 152) were performed using a standard logistic regression approach. All analyses were performed using S-Plus.

### RESULTS

The distribution of allele sizes for the CAG and GGC repeats in *BRCA1/2* carriers, unselected BC cases and controls is presented in Figure 1A and 1B. Mean age at BC onset was 41.6 years for all carriers, 35.6 years in non-Ashkenazi carriers compared with 42.7 years for Ashkenzi mutation carriers. Median CAG repeat length was significantly longer in the Ashkenazi compared with non-Ashkenazi carriers (23 and 21 respectively, 2 sided $P$ = 0.01); at least one allele with repeat length ≥28 was found in 17/154 (11%) of the Ashkenazi carriers, but in none of the 30 non-Ashkenazi carriers (Table 1).

Median CAG repeat length was 23 repeats in carrier BC cases, 22 in ovarian cancer carriers and 21.5 in unaffected carriers. We found no significant evidence of a trend in breast cancer with average CAG repeat length when analysed as a continuous variable (Table 2), regardless of the assumed incidence rates. Using model (b) (incidence rates of 50% of BCLC rates) the estimated relative risk, per repeat unit, was 1.05 (95%CI 0.94–1.17) for breast cancer and 1.00 (95%CI 0.83–1.20) for ovarian cancer. We also conducted analyses restricted to Ashkenazi carriers and analysed *BRCA1* and *BRCA2* carriers separately, but no significant differences were found (data not shown).

12 of the carriers affected with BC (11%) had at least one repeat length of 28 or greater, compared with 2 of the ovarian cancer cases (7%) and 2 of the unaffected carriers (6%). There was no significant effect of the presence of one or two ≥ 28 repeat allele by any of the methods used. With assumption (b), the estimated relative risk of breast cancer was 1.08 (95%CI 0.45–2.61), whilst using the Cox regression approach the relative risk estimate was reduced to 0.89 (95%CI 0.44–1.78). The only subgroup in which there was some suggestion of an effect was in those with *BRCA1* Ashkenazi mutations, where 7/71 (10%) of cases carried at least

**Table 1** Clinical characteristics of carriers of *BRCA1/2* mutations according to ethnic origin and *AR* CAG repeat length

| | **All carriers** | **Non-Ashkenazi**[a] | **Ashkenazi**[a] | |
|---|---|---|---|---|
| | **($n$ = 188)** | **($n$ = 30)** | ***BRCA1***[c] **($n$ = 112)** | ***BRCA2***[c] **($n$ = 45)** |
| **1. All patients** | | | | |
| Breast cancer | 122 (66%) | 19 (63%) | 71 (64%) | 32 (71%) |
| Average age[e] | 41.6 | 35.6 | 41.6 | 45.1 |
| (range)/years | (20–74) | (27.6–47) | (20–73) | (24–52) |
| Unaffected[b] | 65 (35%) | 11(37%) | 41 (37%) | 13 (29%) |
| **2. At least one CAG repeat ≥28**[d] | | | | |
| Breast cancer | 13 (11%) | | 7 (10%) | 5 (16%) |
| Average age/yrs | 47.8 | | 47.11 | 47.8 |
| [b]Unaffected | 4 (6%) | | 2 (5%) | 2 (15%) |
| Average age/yrs | 62.75 | | | |
| Total no. (%) | 17 (9%) | 0 | 10 (9%) | 7 (16%) |

[a]'Ashkenazi origin' includes both Israeli and British carriers of Ashkenazi founder mutations from Ashkenazi origin. [b]Unaffected with breast cancer, ovarian cancer patients included. [c]One carrier of both a *BRCA1* and a *BRCA2* mutation not included. [d]Four individuals without CAG results not included. [e]Age at onset was not recorded for four individuals (all *BRCA1* carriers).

**Table 2** Risk for breast and ovarian cancer in *BRCA1/2* carrier and in non-carrier BC cases associated with (CAG)n and (GGC)n repeats in the *AR* gene

| | **Breast cancer RR (95% CI)** | **Ovarian cancer RR (95% CI)** |
|---|---|---|
| **1. CAG repeat length in *AR* as a continuous covariate** | | |
| *BRCA1/2* carriers (a) | 1.03 (0.94–1.12) | 0.99 (0.82–1.19) |
| (b) | 1.05 (0.94–1.17) | 1.00 (0.83–1.20) |
| COX | 1.01 (0.94–1.09) | 0.98 (0.83–1.16) |
| non-carrier BC cases | 1.00 (0.91–1.10) | |
| **2. ≥ 28 repeats Vs < 28 repeats in AR** | | |
| *BRCA1/2* carriers (a) | 0.89 (0.44–1.78) | 0.44 (0.096–2.03) |
| (b) | 1.08 (0.45–2.61) | 0.58 (0.13–2.59) |
| COX | 0.80 (0.44–1.46) | 0.48 (0.11–2.05) |
| non-carrier BC cases | 1.27 (0.83–1.96) | |
| **3. GGC repeat as a continuous covariate** | | |
| *BRCA1/2* carriers (a) | 0.97 (0.84–1.13) | 0.89 (0.65–1.21) |
| (b) | 0.96 (0.80–1.15) | 0.90 (0.66–1.22) |
| COX | 0.99 (0.87–1.12) | 0.87 (0.67–1.12) |
| non-carrier BC cases | 1.05 (0.90–1.22) | |

RR = Relative risk. COX = Cox regression analysis. (a) Using *BCLC* incidence rates (b) Using 50% of *BCLC* incidence rates

© 2001 Cancer Research Campaign

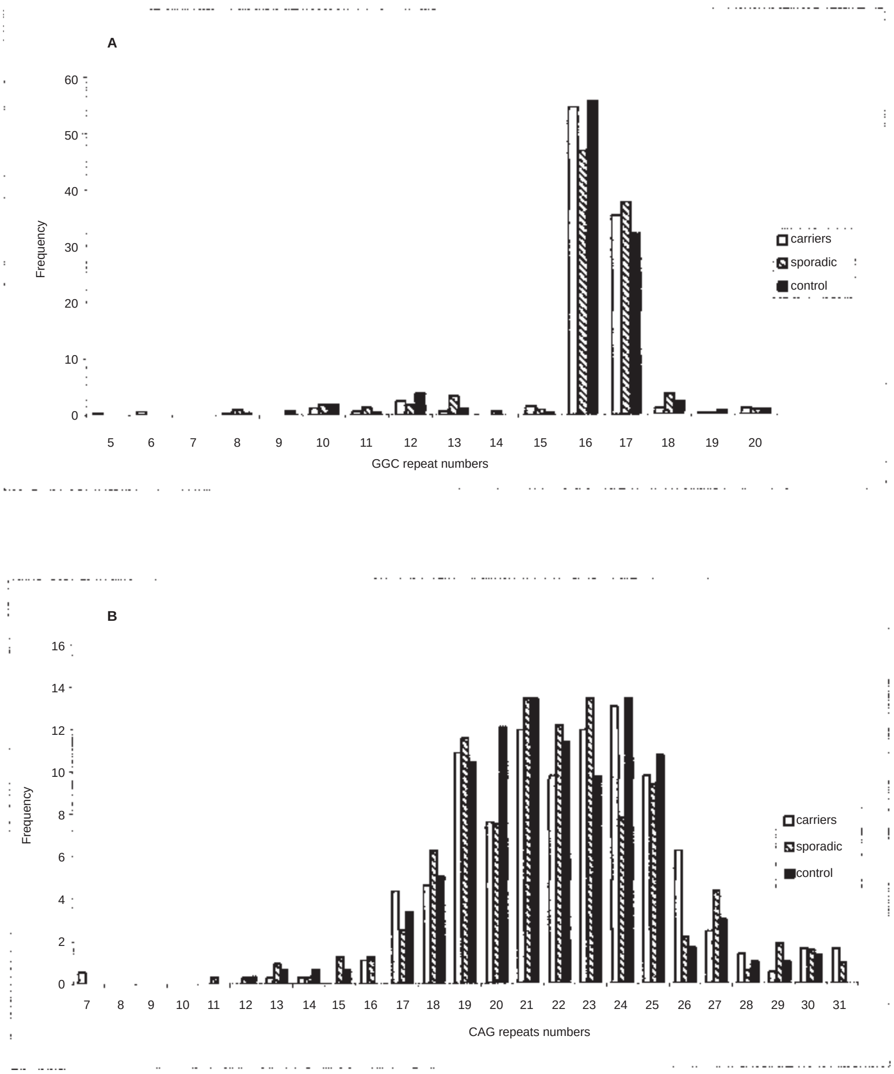

**Figure 1**

one ≥28 repeat allele, compared with 2/41 (5%). However, the mean age of the breast cancer cases with long alleles was significantly older than other affected carriers (47.2 vs 40.4, $P = 0.04$) and the overall estimated relative risks using model (b) was still close to 1.00 (1.15, 95%CI 0.34–3.98).

We found no significant effect of the GGC repeat length on BC and OC risk in carriers, for any of the methods of analysis. There was also no significant difference in the distribution of either CAG or GGC repeat lengths between the non-carriers, BC cases and controls (Figure 1A, 1B and Table 2). The median CAG repeat lengths were 22 in both the cases and controls. Repeat lengths of 28 and over were slightly more common in cases than controls (9% Vs 6%) but again the difference and RR (1.27, 95%CI 0.83–1.96) was not statistically significant. No association of age at BC onset with allele size was found.

### DISCUSSION

Several genes have been suggested as potential modifiers of breast or ovarian cancer risk, both in the general population and in *BRCA1/2* carriers. Given the hormone-dependent nature of breast cancer, genes involved in sex steroid synthesis and metabolism, and steroid hormone receptors, are obvious candidates. The androgen receptor (AR) immunohistochemistry is positive in a high proportion of BC tumours (Isola, 1993; Kuenen et al, 1996). Androgens appear to be associated with growth inhibition in some BC cell lines but a proliferative effect in others. In postmenopausal women high levels of free testosterone elevate the risk for breast cancer possibly through a direct oestrogenic effect or indirectly as a precursor to oestrogen (Sereto et al, 1991; Cauley et al, 1999). The AR pathways might have greater importance in premenopausal women, with an oestrogen-saturated milieu (Adams, 1998). Reports of BC risk associated with androgen levels conducted in this age group, show contradicting results (Buldrook et al, 1977; Sereto et al, 1989). The importance of growth-stimulating versus growth-inhibitory AR effects remains to be elucidated.

The *AR* gene, on chromosome Xq11–12, contains two trinucleotide repeat polymorphisms in exon 1, the (CAG)n codes for polyglutamine (Sleddens et al, 1992) and (GGC)n codes for polyglycine (Sleddens et al, 1993). They are both located in the *N*-terminal domain of the receptor. The $(CAG)_n$ repeat length has been shown to be inversely correlated with ligand-receptor transcription activity (Kazemi-Esfajami et al, 1995). Rare variants with repeat lengths >40 are responsible for X-linked spinal and bulbar muscular atrophy and androgen insensitivity (La Spada et al, 1991). A number of studies have suggested that shorter CAG repeat length is associated with an increased risk of prostate cancer, consistent with a higher level of androgen stimulation to the prostate associated with a more active receptor (Hakimi et al, 1997; Ingles et al, 1997). However, not all prostate cancer case-control studies have been able to replicate this effect (Edwards et al, 1999). The functional significance of the (GGC)n is unknown.

Our study, based mainly on subjects from the Ashkenazi Jewish population, failed to find any significant association of the AR repeat polymorphisms with risk of BC or ovarian cancer in either *BRCA1/2* carriers or in non-carriers. Rebbeck et al (1999) estimated a relative risk of 1.81 (95%CI 1.06–3.08) for breast cancer in *BRCA1* carriers with at least one ≥28 repeat allele. Using the same method of analysis (Cox regression) our upper 95% confidence interval excludes their estimated relative risk. As discussed above this method does not seem appropriate (at least for relative risk estimation) when carriers have been ascertained on the basis of disease status and age. However, using our preferred method, the results are qualitatively similar, although the confidence limits on the relative risk are wider.

Our study included data on both *BRCA1* and *BRCA2* carriers. Combining data on both *BRCA1* and *BRCA2* mutation carriers seems reasonable, given that both types of mutation confer similar breast cancer risks, and that the functions of the two genes appear to be similar. However, when restricting analysis to *BRCA1* carriers (or to *BRCA2* carriers) we found a similarly negative result, although the confidence intervals were wider. We also combined data on both Ashkenazi and non-Ashkenazi carriers. Since the distribution of CAG repeat length differed between populations (Edwards et al, 1992), and the distribution of disease status and age at onset also differed (with non-Ashkenazi cases being younger) it was necessary to correct for population in the analysis by stratification. We did find a significantly higher age at onset amongst Ashkenazi *BRCA1* carriers with at least one ≥28 repeat alleles. However, since the proportion of affected carriers was lower in the group with a ≥28 repeat allele, the estimated RR was still close to 1.00 and non-significant.

A recent study by Yu et al (1999) suggested an association between shorter CAG repeat lengths and tumour grade and survival. In our study, and in other studies of carriers, affected carriers will be biased towards those with longer survival, and this could introduce an artefactual association between repeat length and disease. This effect is likely to be small, however future studies based on incident cancers could avoid this bias.

In conclusion, we find that *AR* repeat length does not have a substantial effect on breast or ovarian cancer risk, either in *BRCA1* or *BRCA2* carriers, or in the general population. A larger study would be required to detect a more moderate effect.

### ACKNOWLEDGEMENTS

LK has a Barclay fellowship through the British Council. We thank the Radlett Synagogue Community for its support for this study.

DE/SE are supported by the Cancer Research Campaign. ZKJ is supported by The Prostate Cancer Charitable Trust. FD is supported by a Burroughs Wellcome Fellowship.

### REFERENCES

Adams JB (1998) Adrenal androgen and human breast cancer: a new appraisal. *Breast Can Res Treat* **51**: 183–188

Buldrook RD, Hayward JL and Spicer CC (1971) Relation between urinary androgen and corticosteroids excretion and subsequent breast cancer. *Lancet* **1**(2): 395–397

Cauley JA, Lucas FL, Kuller LH, Stone K, Browner W and Cummings SR (1999) Elevated serum estradiol and testosterone concentration are associated with a high risk for breast cancer. *Ann Intern Med* **130**: 270–277

Dunning AM, McBride S, Gregory J, Durocher F, Foster NA and Healey CS, et al (1999) No association between androgen or vitamin D receptor gene polymorphisms and risk of breast cancer. *Carcinogenesis* **20**: 2131–2135

Easton DF, Ford D and Bishop DT. Breast Cancer Linkage Consortium (1995) Breast and ovarian cancer incidence in *BRCA1* mutation carriers. *Am J Hum Genet* **56**: 265–271

Edwards A, Hammond HA and Jin I, et al (1992) Genetic variation in five trimeric and tandem repeat loci in four human population groups. *Genomics* **12**: 241

Edwards SM, Bazioch MD, Minter R, Hamoudi R, Collins N and Arden-Jones A, et al (1999) Androgen receptor polymorphisms: association with prostate cancer risk, relapse and overall survival. *Int J Cancer* **84**: 458–465

Ford D, Easton DF, Stratton MR, Narod S, Goldgar D and Devilee P, et al (1998) Genetic heterogeneity and penetrance analysis of *BRCA1* and *BRCA2* genes in breast cancer families. *Am J Hum Genet* **62**: 676–689

Hakimi JM, Schoenberg MP, Rondinelli RH, Piantadosi S and Barrack ER (1997) Androgen receptor variants with short glutamine or glycine repeats may identify unique subpopulation of men with prostate cancer. *Clin Cancer Res* **3**: 1599–1608

Ingles SA, Ross RK, Yu MC, Irvine RA, La Pera G, Haile RW and Coetzee GA (1997) Association of prostate cancer risk with genetic polymorphisms in vitamin D receptor and androgen receptor. *J Natl Cancer Inst* **89**: 166–170

Isola JJ (1993) Immunohistochemical demonstration of androgen receptor in breast cancer and its relationship to other prognostic factors. *J Pathol* **170**: 31–35

Kazemi-Esfajami P, Trifiro MA and Pinsky L (1995) Evidence for a repressive function of the long polyglutamine tract in the human androgen receptor: possible pathogenic relevance for the (CAG)n expanded neuropaties. *Hum Mol Genet* **4**: 523–527

Kuenen Boumeester V, Van der Kwast TH, Claassen CC, Look MP, Lien GS, Klijn JG and Henzen Longmans SC (1996) The clinical significance of androgen receptor in breast cancer and its relation to histological and cell biological parameters. *Eur J Cancer* **32A**: 1560–1565

La Spada AR, Wilson EM, Lubahn DB, Harding AE and Fischbeck KH (1991) Androgen receptor gene mutations in X-linked spinal and bulbar muscular atrophy. *Nature* **352**: 77–79

Peto J, Collins N, Barfoot R, Seal S, Warren W and Rahman N (1999) Prevalence of *BRCA1* and *BRCA2* gene mutations in patients with early-onset breast cancer. *J Natl Cancer Inst* **91**: 943–949

Rebbeck TR, Kantoff PW, Krithivas K, Neuhausen S, Blackwood MA and Godwin AK (1999) Modification of *BRCA1*-associated breast cancer risk by polymorphic androgen-receptor CAG repeat. *Am J Hum Genet* **64**: 1371–1377

Sereto G, Toniolo P and Pisani P, et al (1989) Androgen and breast cancer in premenopausal women. *Cancer Res* **49**: 471–476

Sereto G, Toniolo P and Berrino F (1991) Serum and urinary androgens and the risk of BC in postmenopausal women. *Cancer Res* **51**: 2572–2576

Sleddens HF, Oostra BA, Brinkmann AO and Trapman J (1992) Trinucleotide repeat polymorphism in the human androgen receptor gene (*AR*). *Nuc Acids Res* **20**: 1427

Sleddens HF, Oostra BA, Brinkmann AO and Trapman J (1993) Trinucleotide (GGN) repeat polymorphism in the human androgen receptor (AR) gene. *Hum Mol Genet* **2**: 493

Spurdle AB, Dite GS, Chen X, Mayne CJ, Southey MC and Batten LE, et al (1999) Androgen receptor exon 1 CAG repeat length and breast cancer in women before age forty years. *J Natl Cancer Inst* **91**: 961–966

Struewing JP, Hartge P, Wacholdes S, Baker SM, Berlin M and McAdams M, et al (1997) The risk of cancer associated with specific mutations of *BRCA1* and *BRCA2* among Ashkenazi Jews. *New Eng J Med* **336**: 1401–1408

Thorlacius S, Struewing JP, Hartge P, Olafsdottir GH, Sigvaldasson H and Tryggvadottir L, et al (1998) Population-based study of risk of breast cancer in carriers of *BRCA2* mutations. *Lancet* **352**: 1337–1339

Yu H, Bharaj B, Giani M and Diamandis EP (1999) CAG repeat of the androgen receptor gene and its association with survival. *Proc Am Ass Cancer Res* **40**: 2518

© 2001 Cancer Research Campaign
